# Sunny Holidays before and after Melanoma Diagnosis Are Respectively Associated with Lower Breslow Thickness and Lower Relapse Rates in Italy 

**DOI:** 10.1371/journal.pone.0078820

**Published:** 2013-11-04

**Authors:** Sara Gandini, Esther De Vries, Giulio Tosti, Edoardo Botteri, Giuseppe Spadola, Patrick Maisonneuve, Chiara Martinoli, Arjen Joosse, Pier Francesco Ferrucci, Federica Baldini, Emilia Cocorocchio, Elisabetta Pennacchioli, Francesco Cataldo, Barbara Bazolli, Alessandra Clerici, Massimo Barberis, Veronique Bataille, Alessandro Testori

**Affiliations:** 1 Division of Epidemiology and Biostatistics, European Institute of Oncology, Milan, Italy; 2 Department of Public Health, Erasmus University Medical Center, Rotterdam, The Netherlands; 3 Division of Melanoma and Muscle-Cutaneous Sarcomas, European Institute of Oncology, Milan, Italy; 4 Department of Medical Oncology, European Institute of Oncology, Milan, Italy; 5 Department of Pathology and Laboratory Medicine, European Institute of Oncology, Milan, Italy; 6 Twin Research and Genetic Epidemiology Unit, King’s College, London, United Kingdom; University of Tennessee, United States of America

## Abstract

**Background:**

Previous studies have reported an association between sun exposure and improved cutaneous melanoma (CM) survival. We analysed the association of UV exposure with prognostic factors and outcome in a large melanoma cohort.

**Methods:**

A questionnaire was given to 289 (42%) CM patients at diagnosis (Group 1) and to 402 CM patients (58%) during follow-up (Group 2). Analyses were carried out to investigate the associations between sun exposure and melanoma prognostic factors and survival.

**Results:**

Holidays in the sun two years before CM diagnosis were significantly associated with lower Breslow thickness (p=0.003), after multiple adjustment. Number of weeks of sunny holidays was also significantly and inversely associated with thickness in a dose-dependent manner (p=0.007). However when stratifying by gender this association was found only among women (p=0.0004) the risk of CM recurrence in both sexes was significantly lower in patients (n=271) who had holidays in the sun after diagnosis, after multiple adjustment including education: HR=0.30 (95%CI:0.10-0.87; p=0.03) conclusions: Holidays in the sun were associated with thinner melanomas in women and reduced rates of relapse in both sexes. However, these results do not prove a direct causal effect of sun exposure on survival since other confounding factors, such as vitamin D serum levels and socio-economic status, may play a role. Other factors in sun seeking individuals may also possibly affect these results.

## Introduction

The incidence of CM has steadily increased over the last 30 years in most fair-skinned populations even if the great majority of the increase has been linked to the increase in diagnoses of thin lesions with excellent prognosis[1]. One of the hypotheses for the discrepancy between incidence and mortality trends is that melanomas are detected at earlier stages in women than in men[2]. Another hypothesis is that part of melanoma epidemic is made of non life-threatening melanomas that could be promoted by sun exposure. Recent sun exposure could be able to trigger skin cancers with little malignant potential. Long noted is the relationship between sun exposure and non-melanoma skin cancer (NMSC) and superficial spreading melanoma (SSM), which are often not aggressive[3].

This last hypothesis arose from the results of a study evaluating the association of sun exposure indicators with melanoma mortality. This American study of 528 melanoma cases showed that markers of sun exposure were inversely associated with death from melanoma[4].In this study, we investigated if different indicators of UV exposure, collected before and after CM diagnosis, are associated with Breslow thickness and recurrence in Italy. We wanted to investigate if there could be an induction period for the most aggressive melanoma.

## Material and Methods

A hospital-based study of melanoma cases was initiated in 2007 at the European Institute of Oncology in Milan, Italy, which is a secondary and tertiary centre for CM diagnosis and treatment. A self-administered questionnaire was given systematically to all patients during clinics (response rate 99%) and data was collected on socio-demographic variables, season of diagnosis and CM site. Skin type was assessed with the Fitzpatrick classification. 

A total of 742 CM patients with histologically confirmed diagnosis of primary CM without relapse were identified via two databases: the Institutional Melanoma Database and the Tumour Registry of the European Institute of Oncology (IEO), Milan, after informed consent was obtained. Use of the data included in The IEO Tumour Registry was approved by the IEO Institutional Review Board (March 2013). Main clinical information and information on disease history of the patients are obtained from the Tumor Registry. The European Institute of Oncology is a tertiary referral centre and assessment of diagnosis, cancer characteristics and recurrence was made by highly qualified dermatologists, pathologists, surgeons and oncologists. 

**Figure 1:**
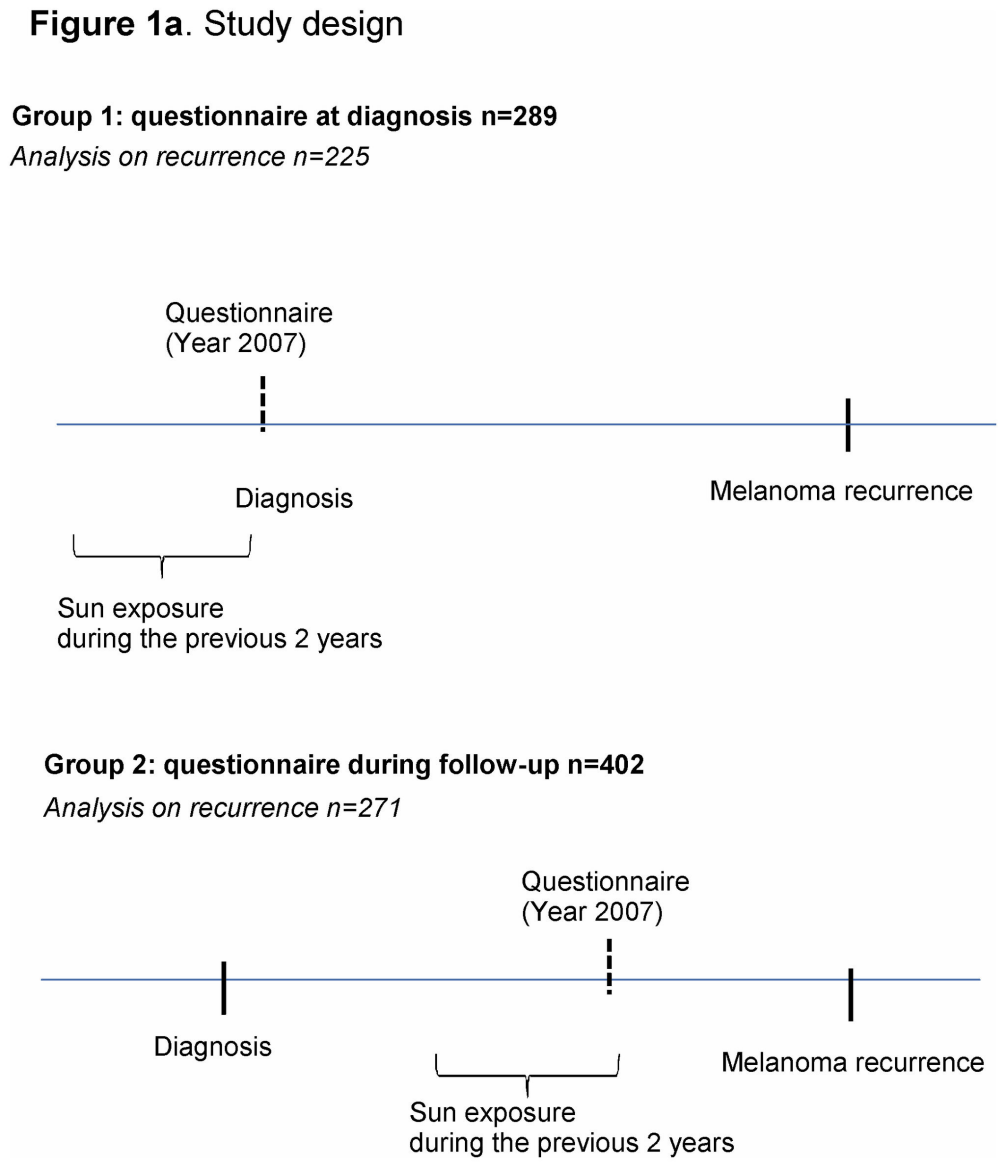
Study design.

As shown in Figure 1a, there were two cohorts of patients which did not overlap: cases interviewed at initial diagnosis (Group 1) and those during follow-up (Group 2), with the same questionnaire. In the latter group, median time from CM diagnosis to questionnaire was 2.6 years (1-6 years inter-quartile range). Sun exposure data for Group 1 was collected by asking CM patients if they had holidays in the 2 years preceding their melanoma diagnosis and if yes, how many weeks of holidays per year for each year. For Group 2 at follow up, they were asked the same questions for a period of 2 years before the questionnaire. We asked information on the last 2 years because this is easy to remember and usually these types of habits before diagnosis do not change. After diagnosis patients will likely change behaviour[5][6][7] and we made the same questions for the Group 2. Data on sun exposure during peak hours (11:00AM-1:00PM) in the previous two years, sunbed exposure (current use and use before age 30) and residence of at least one year in tropical countries before age of 14 was also collected. We used ‘sunny holidays’ as indicator of high sun exposure because Italians sunbath mainly in holidays and it is a measure less prone to recall bias. In fact questions commonly used to measure sun exposure, especially those reflecting past exposures, embody attempts to recall events that are subject to both systematic recall bias and random misclassification[8][9]. Recent sunny holidays will be less subject to misclassification. Furthermore recall bias characterize case-controls designs and in our study design comparing melanoma cases this information should be more reliable. Sunny holidays will be referred to holidays in the manuscript. 

Patients with previous CM primary, missing histology, in situ, acral melanoma, mucosal melanoma, vulvar or ano-rectal melanoma and patients with distant metastases at diagnosis were excluded (n=51, 7%), leaving 691 patients eligible for the study: 289 were interviewed at diagnosis (42%, Group 1) and 402 during follow-up (58%, Group2). 

For the analyses on CM recurrence, 496 patients with pT1-T3N0M0 staging were included: 225 interviewed at diagnosis (group 1) and 271 after diagnosis (group 2). For group 2 we included patients with a diagnosis of CM at the most 7 years before the questionnaire, to avoid inclusion of very long survivors. No residents in other countries were found. Questions on sun exposure refer to two years before diagnosis for group 1 and for group 2, for 2 years before the questionnaire during follow-up. In order to avoid time related bias, the competing risk analysis in group 2 started from the time of interview (free of disease). A sensitivity analysis was carried out including also long survivors. 

### Statistical methods

Associations between categorical variables at baseline and median Breslow thickness was evaluated by non-parametric median two sample tests. Associations between categorical variables and holidays were evaluated by the Chi-square test, Fisher exact test and the Chi-square test for trend, as appropriate. 

In multivariate analyses, Breslow thickness was either analysed as categorical variable or as continuous variable. Logistic models were used to evaluate the probability to have a thick CM (Breslow >1mm) and an ANCOVA model was introduced, transforming Breslow thickness for normal distribution. All possible confounding factors were evaluated in multivariate models.

For cumulative incidence, only first events of interest were considered. For the cumulative incidence of CM-related events, CM-related recurrences from a primary CM and deaths were counted as events, while second CM, NMSC and non-CM primary cancers were considered as competing events, since they may be associated to a genetic predisposition or other causes. Follow-up of patients free of events was censored at the date of last visit. Cumulative incidences were compared across groups by means of the Gray test. Multivariable Cox proportional hazards models were applied to evaluate holidays as independent prognostic factor and assumptions on proportional hazards were verified. Adjusted hazard ratios (HR) with 95% confidence intervals (CIs) were reported. All analyses were carried out with SAS software version 9.2 (SAS Institute, Cary, NC) and R software, version 2.12.2 (http://www.r-project.org). All reported p values were two-sided.

## Results

Six hundred ninety-one histologically confirmed primary CM, diagnosed between 1984 and 2012 were eligible for the study. Median age at diagnosis was 47 years (inter-quartile range: 37–60), 375 (54%) had a thick CM (Breslow>1mm), 378 (55%) were women, 519 (75%) had a high level of education (high school at least) and 449 (65%) lived in Northern Italy. Seventy-three percent (n=501) had a superficial spreading melanoma (SSM) and 15% (n=101) nodular melanoma (NM). All potential factors associated with Breslow thickness are shown in Table 1. 

**Table 1:** Breslow’s thickness by socio-demographic characteristics and clinical features.

**Feature**	**Categories**	**N**	**Median Breslow thickness (IQR)**	**P-value1**
**Gender**	Female	378	1.0 (0.5, 1.9)	0.001
	Male	313	1.3 (0.7, 2.4)	
**Age>60 years**	Yes	159	1.6 (0.8, 3.0)	<.0001
	No	532	1.0 (0.6, 2.0)	
**Education2**	At least high school	519	1.0 (0.5, 1.9)	<.0001
	No High school	172	1.5 (0.9, 3.0)	
**Residence3**	Northern Italy	449	1.2 (0.6, 2.4)	0.136
	Central/southern Italy	242	1.0 (0.6, 2.1)	
**BMI>25**	Yes	291	1.3 (0.6, 2.7)	0.003
	No	400	1.0 (0.6, 1.9)	
**Phototype**	I-II	364	1.2 (0.7, 2.2)	0.029
	III-IV	314	1.0 (0.5, 2.1)	
	Missing	13	2.2 (1.1, 4.0)	
**Current smoker**	Yes	297	1.2 (0.6, 2.4)	0.17
	No	383	1.0 (0.6, 2.0)	
	Missing	11	1.1 (0.6, 3.8)	
**Medical visit at diagnosis**	Dermatologist	321	1.0 (0.6, 1.8)	0.002
	Other medical doctor	106	1.5 (0.6, 3.0)	
	Missing	264	1.1 (0.6, 2.3)	
**Season of diagnosis**	Summer	188	1.2 (0.7, 2.2)	0.161
	Autumn	182	1.0 (0.5, 2.0)	
	Winter/springtime	321	1.1 (0.6, 2.3)	
**Histological type**	SSM	501	0.9 (0.5, 1.5)	<0.0001
	NM	101	3.0 (2.1, 4.3)	
	Other	5	2.3 (2.1, 3.0)	
	Missing	84	1.7 (0.7, 3.0)	
**CM family history**	Yes	35	1.0 (0.6, 2.2)	0.324
	No	458	1.2 (0.7, 2.3)	
	Missing	198	1.0 (0.5, 1.9)	
**NMSC personal history**	Yes	17	1.1 (0.7, 2.0)	0.927
	No	674	1.1 (0.6, 2.2)	
**Other cancer personal history**	Yes	15	1.2 (0.9, 2.5)	0.425
	No	676	1.1 (0.6, 2.2)	

P-value from Wilcoxon test, excluding missing values.

BMI: Body Mass Index; CM: Cutaneous Melanoma; NMSC: Non-Melanoma Skin Cancer. SSM: Superficial Spreading Melanoma. NM: Nodular melanoma

As expected men, older cases (above 60 years) and those with low levels of education had thicker CM compared to women, younger and more educated patients, respectively (Table 1). CMs diagnosed by a dermatologist had a lower Breslow thickness compared to those detected by general practitioners or other specialists (median Breslow 1.0 vs.1.5 mm; p=0.002). The female frequency of thick CM was significantly lower for high education than in low education (40% vs 71%; p<0.0001), whereas male thickness in men did not change by education (Figure 1b). In multivariable logistic models assessing the probability of thick (> 1mm) vs. thin (≤1mm) CM, after adjusting for age, gender, grade of clinician, BMI, family history and personal history of NMSC, the interaction between thickness and education in women remained significant (p=0.03): a 77% reduction in risk of thick CM for highly educated women (OR=0.23; 95%CI: 0.12 to 0.45) compared to low educated women, whereas this was not significant in men (OR=0.67; 95%CI: 0.33 to 1.36).

Frequencies of socio-demographic, clinical features and UV exposure indicators categorised by holidays, for the 289 patients evaluated at diagnosis, are presented in Table 2. Patients with higher level of education went more often on holidays than those with low educational levels (83% vs 65%; p<0.0001). Ulcerated CM and CM diagnosis during the summer were more common in those without holidays (Table 2). Slightly younger subjects were observed in those having holidays but this did not reach significance (p=0.07). 

**Table 2:** Proportion of patients by sunny holidays at diagnosis (Group 1). n (%).

	**Categories**	**Sunny holidays**	**No Sunny holidays**	
		206 (100%)	83 (100%)	P-value*
**Gender**	Male	101 (49.0)	43 (51.8)	0.669
	Female	105 (51.0)	40 (48.2)	
**Age at diagnosis**	≤ 60 years	162 (78.6)	57 (68.7)	0.074
	> 60 years	44 (21.4)	26 (31.3)	
**Residence**	Northern Italy	140 (68.0)	63 (75.9)	0.181
	Southern Italy	66 (32.0)	20 (24.1)	
**Education**	At least high school	172 (83.5)	54 (65.1)	<0.001
	No High school	34 (16.5)	29 (34.9)	
**Phototype**	I-II	114 (55.3)	43 (51.8)	0.651
	III-IV	87 (42.2)	37 (44.6)	
	missing	5 (2.4)	3 (3.6)	
**Breslow**	≤1.0	97 (47.1)	29 (34.9)	0.016
	1.1-2.0 mm	55 (26.7)	19 (22.9)	
	2.1-4.0 mm	37 (18.0)	18 (21.7)	
	>4	17 (8.3)	17 (20.5)	
**Lymph node involvement**	Yes	16 (7.8)	11 (13.3)	0.147
	No	190 (92.2)	72 (86.7)	
**Ulceration**	Yes	38 (18.4)	24 (28.9)	0.049
	No	155 (75.2)	54 (65.1)	
	Missing	13 (6.3)	5 (6.0)	
**Histology**	SSM	165 (80.1)	60 (72.3)	0.225
	NM	24 (11.7)	16 (19.3)	
	Other	2 (1.0)	1 (1.2)	
	missing	15 (7.3)	6 (7.2)	
**Family history of melanoma**	Yes	9 (4.4)	3 (3.6)	0.951
	No	137 (66.5)	55 (66.3)	
	Missing	60 (29.1)	25 (30.1)	
**Personal history of NMSC**	Yes	4 (1.9)	5 (6.0)	0.071
	No	202 (98.1)	78 (94.0)	
**Grade of clinician at diagnosis**	Dermatologist	126 (61.2)	48 (57.8)	0.112
	Other doctor	49 (23.8)	15 (18.1)	
	Missing	31 (15)	20 (24.1)	
**Melanoma on sun exposed site**	Yes	40 (19.4)	15 (18.1)	0.827
	No	166 (80.6)	67 (80.7)	
	Missing	0 (0.0)	1 (1.2)	
**Holidays in tropical countries**	Yes	60 (29.1)	16 (19.3)	0.072
	No	138 (67)	65 (78.3)	
	Missing	8 (3.9)	2 (2.4)	
**Sun exposure during peak hours (11:00AM- 1:00PM)**	Yes	103 (50.0)	20 (24.1)	<0.001
	No	99 (48.1)	59 (71.1)	
	Missing	4 (1.9)	4 (4.8)	
**Sunbed (current user)**	Yes	18 (8.7)	8 (9.6)	0.813
	No	185 (89.8)	74 (89.2)	
	Missing	3 (1.5)	1 (1.2)	
**Sunbed use before age of 30**	Yes	48 (23.3)	21 (25.3)	0.759
	No	153 (74.3)	61 (73.5)	
	Missing	5 (2.4)	1 (1.2)	
**Season of diagnosis**	Summer	40 (19.4)	32 (38.6)	0.001
	Autumn	55 (26.7)	12 (14.5)	
	Winter/springtime	111 (53.9)	39 (47.0)	

* P-value from Chi-square or Mantel-Haenszel Chi-Squares for trend, excluding missing values CM: Cutaneous Melanoma; NMSC: Non-Melanoma Skin Cancer. SSM: Superficial Spreading Melanoma. NM: Nodular melanoma.

**Figure 2:**
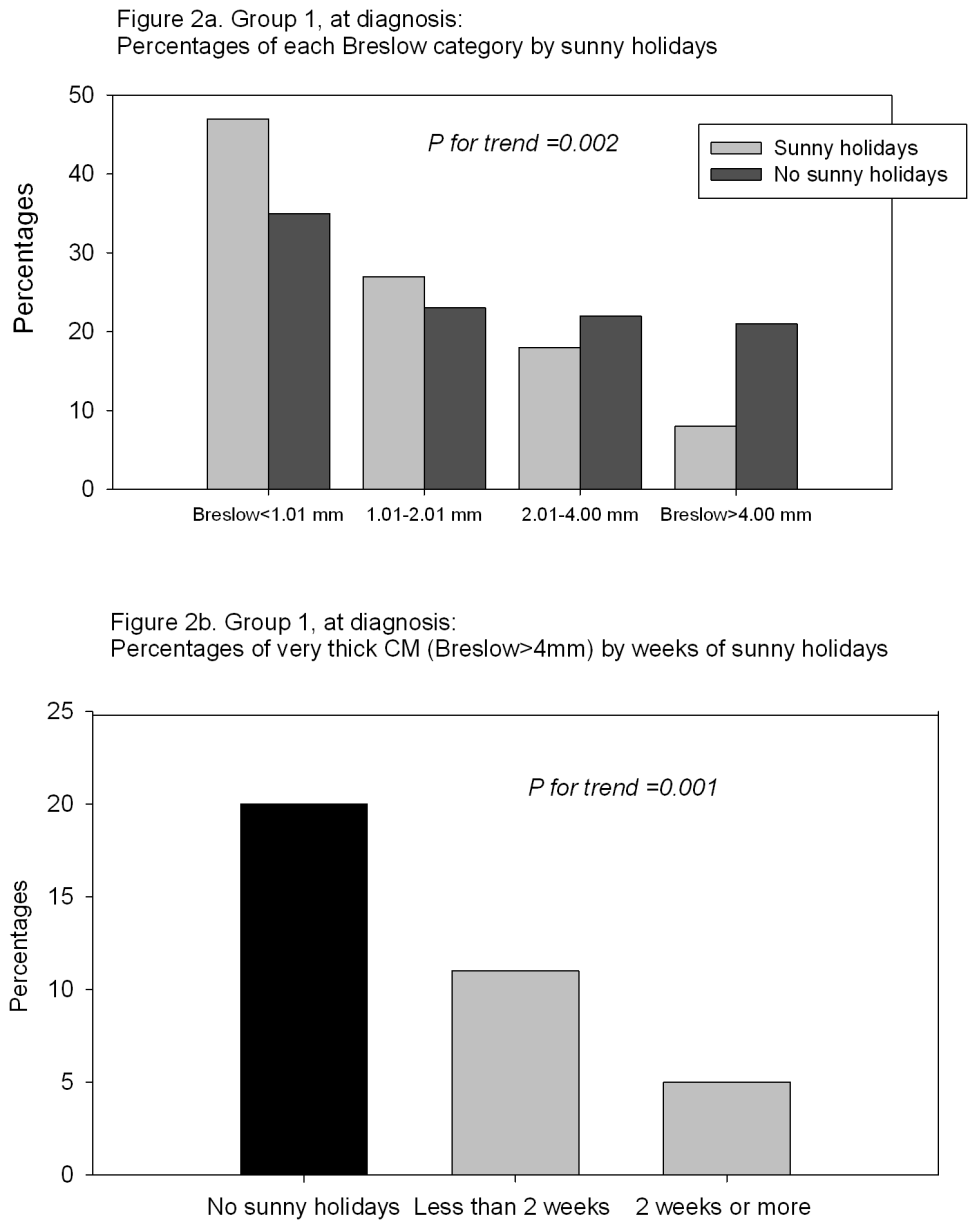
Group 1, at diagnosis. A) Percentages of each Breslow category by sunny holidays. B) Percentages of very thick CM (Breslow>4mm) by weeks of sunny holidays.

Breslow categories (pT) were associated with holidays: the proportion of very thick CM (>4 mm Breslow thickness) was significantly lower among patients having holidays (8% for holidays vs 20% for no holidays; p for trend=0.002; Figure 2a). Very thick melanoma were also negatively associated with number of weeks of holidays (categorized as ‘no sunny holidays’, ‘1-2 weeks of sunny holidays per year’, and ‘>2 weeks per year’) in a dose response manner (p for trend=0.001; Figure 2b). 

**Figure 3:**
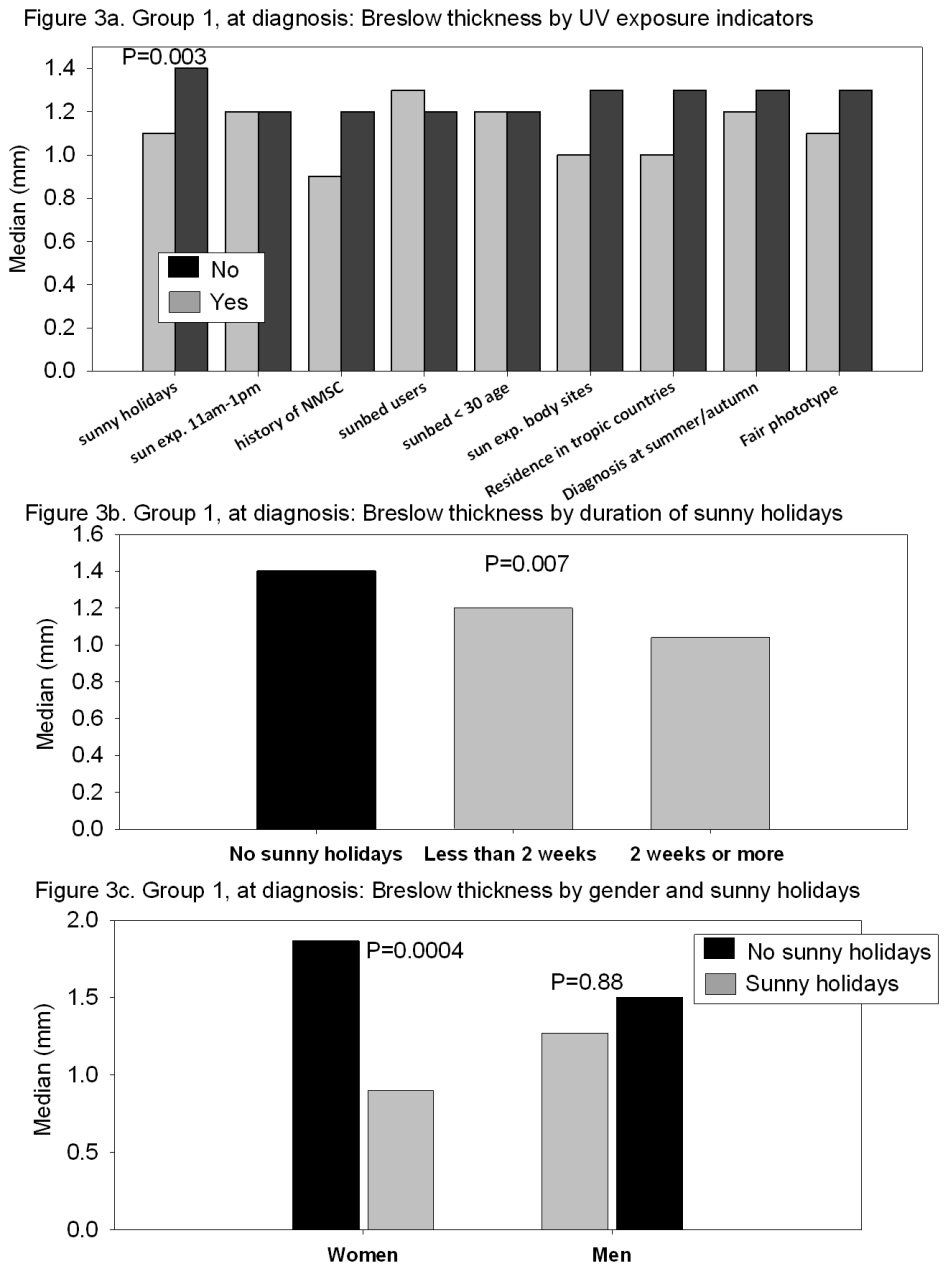
Group 1, at diagnosis. A) Breslow thickness by UV exposure indicators. B) Breslow thickness by duration of sunny holidays. C) Breslow thickness by gender and sunny holidays.

Median Breslow thickness values are presented in Figure 3a for several categorical variables possibly associated with UV exposure. After adjusting for several confounding factors (age, gender, education, grade of clinician at visit, history of NMSC and season at diagnosis) holidays before diagnosis remained significantly associated with lower Breslow thickness (p=0.003). Exposure during peak hours, history of NMSC, sunbed use and CM body site, were not significantly associated with Breslow thickness in multivariate analyses, neither were skin type nor season of CM diagnosis. 

Frequencies of sunny holidays were significantly lower in the group evaluated during follow-up (72% vs. 62% for Group1 and Group 2 respectively; P=0.01) compared to the group at diagnosis as well as sun exposure during peak hours (44% vs. 27%; P<0.0001), whereas sunbed exposure did not change (11% vs. 10%; p=0.28). 

Holidays were also significantly associated with Breslow thickness in a dose-response manner (p=0.007; Figure 3b). We found a significant interaction between the effect of holidays and gender (p=0.004; Figure 3c): women had significantly lower Breslow thickness if they had a history of holidays (p=0.0004), whereas for men this protective effect of sun exposure was not significant (p=0.88). 

For CM recurrence, median follow-up was 44 months (range 1-72) for group 1 and 40 months (range 2-75) for group 2. Melanoma staging was very similar according to holidays, as well as when comparing group 1 and 2 (Table 3). 

**Table 3:** Events among low stage melanoma patients (pT1-T3N0M0) by sunny holidays.

			**Sunny holidays** n=341	**No sunny holidays** n=155	**P-value**	**HR (95%CI)**
**Group 1**	**Baseline**	Breslow	1.0 (0.5, 1.7)	1.0 (0.6, 2.1)	0.59‡	
**n=225**		Median (Q1, Q3)				
		Ulcerated	26 (17%)	11 (20%)	0.57†	
	**At follow-up**					
		CM recurrence	11 (7%)	1 (2%)	0.18¥	4.19 (0.53-33.36)
					(0.64^§^)	
		Second primary NMSC	3 (2%)	1 (2%)		
		Second primary CM	5 (3%)	1 (2%)		
		Other cancer	2 (1%)	1 (2%)		
		Death	3 (2%)	2 (4%)	0.28¥	
**Group 2**	**Baseline**	Breslow	0.9 (0.5, 1.5)	1.0 (0.5, 1.7)	0.57‡	
**n=271**		Median (Q1, Q3)				
		Ulceration	14 (9%)	13 (15%)	0.19†	
	**At follow-up**					
		CM recurrence	9 (5%)	10 (10%)	0.03¥	0.30 (0.10-0.87)*
					(0.07§)	
		Second primary NMSC	2 (4%)	0 (1%)		
		Second primary CM	8 (5%)	3 (3%)		
		Other cancer	2 (1%)	0 (0%)		
		Death	3 (2%)	2 (2%)	0.38¥	

* In group 2 very long survivors were excluded. ¥Cox model adjusted for gender, Breslow thickness, ulceration and degree of doctor. § Gray test for competing risk analysis. ‡ Wilcoxon tests for median values; † Chi-square/Fisher exact tests. CM: Cutaneous Melanoma; NMSC: Non-Melanoma Skin Cancer.

Overall, six percent of patients had a melanoma recurrence and 5% a second primary cancer (Table 3). 

**Figure 4:**
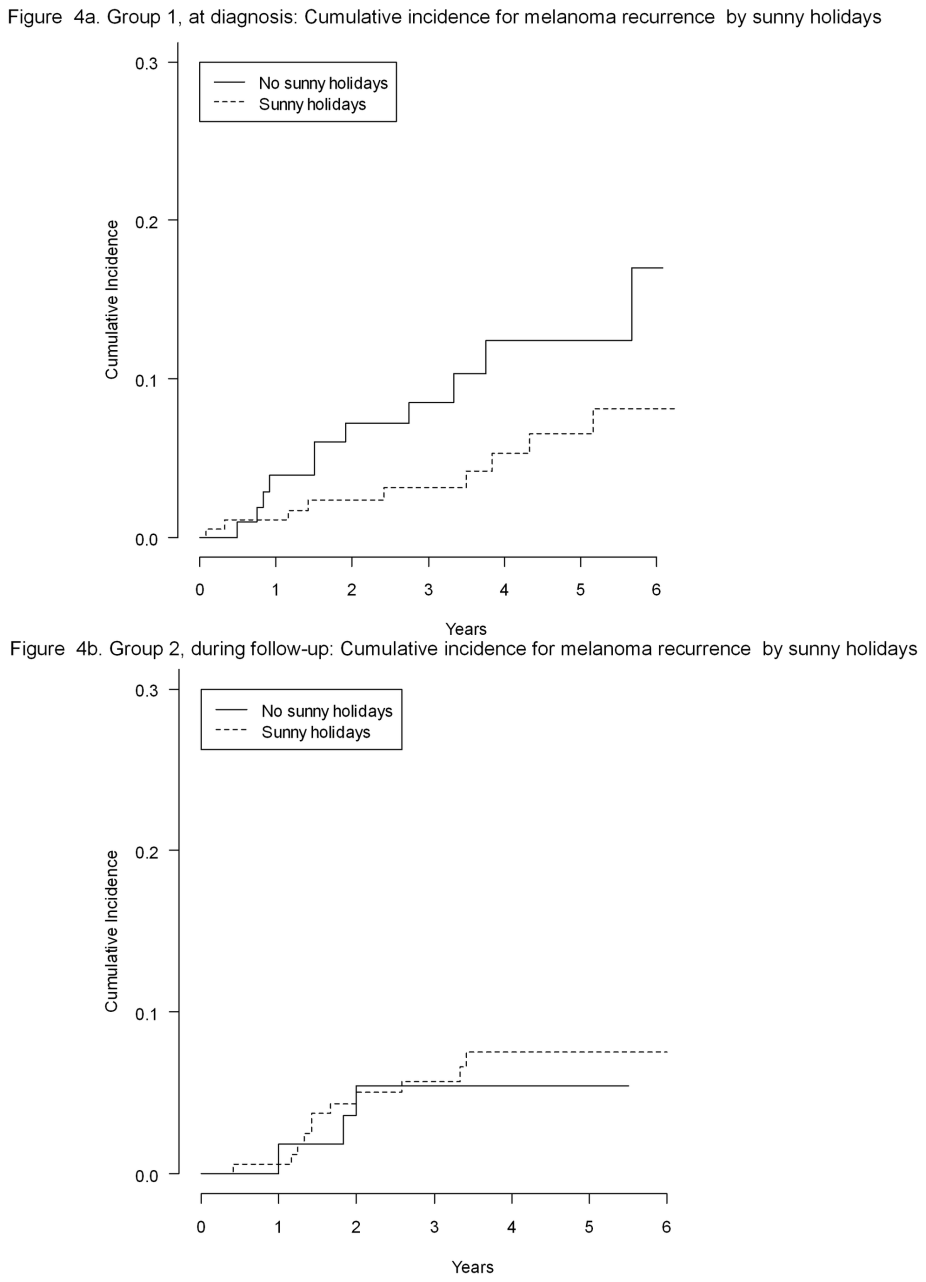
Group 1, at diagnosis: cumulative incidence for melanoma recurrence by sunny holidays (A). Group 2, during follow-up: cumulative incidence for melanoma recurrence by sunny holidays.

Holidays before diagnosis was not associated with risk of recurrence (p=0.64 Gray test; HR=4.19; 95%CI: 0.53 to 33.36 with p=0.18; Figure 4a). However, a significant trend was found when looking at holidays during follow-up. The 5-year cumulative incidence of CM recurrences was 8% for those having holidays after diagnosis compared to 17% for those without (p=0.07 Gray test; Figure 4b). After adjustment for gender, Breslow thickness, ulceration and grade of clinician, the beneficial impact of holidays on relapse after diagnosis was statistically significant: HR of 0.30 (95%CI: 0.10 to 0.87; p=0.03). The results were not affected by further adjustment for age, education and lag time from diagnosis to questionnaire. 

Median time from diagnosis to questionnaire in group 2 was 28.8 for those having holidays after diagnosis compared to 29.1 months for those not having holidays respectively. Including long survivors the difference in recurrence between exposed and unexposed is even more significant (HR=0.24, 95%CI: 0.07-0.75 and P=0.01). When restricting the analyses for those with no more than 5 years from diagnosis to questionnaire within group 2, the hazard ratio still indicated a 60% reduction in risk of recurrence, even if it lost statistical significance: HR=0.38 (95%CI: 0.12 to 1.23; p=0.12). 

We also found a dose-response relationship between risk of melanoma recurrence and number of weeks of holidays. The hazard ratio for up to 2 weeks or more of holidays were: HR=0.74 (95%CI: 0.16 to 3.45) and HR=0.28 (95%CI: 0.08 to 0.98), respectively, compared to patients not going on holidays.

## Discussion

A history of holidays in the sun for 2 years before CM diagnosis was significantly associated with lower Breslow thickness with a significant dose response in Italy. Sunny holidays after CM diagnosis were also associated with lower rates of CM recurrence. No association was found with sunbed exposure and sun exposure during peak-hours and we observed a lower frequencies of sunny holidays after melanoma diagnosis. Our results may possibly be explained by Vitamin D serum levels. However, the observed protective effect of sun exposure in relation to CM thickness reached statistical significance only among women. Vitamin D serum levels have been reported to be lower in women compared to men even in a sunny country like Italy[10].Thinner CM and lower recurrence rates have previously been linked to higher vitamin D serum levels[11].Other observational and case-control studies found a beneficial effect of vitamin D serum levels on melanoma survival[12-14].It has also been hypothesised that Vitamin D might have a beneficial influence for total mortality and incidence of different types of cancers, so this does not apply to melanoma only[15-20].The effect of sun exposure was independent from known melanoma prognostic factors as well as skin awareness or screening indicators. 

High educational level, that can be considered a proxy for socio-economic status, was found to be significantly associated with thin CM but only among women. A limitation of this study is the lack of information on types of occupation (e.g. indoor versus outdoor work) and socio-economic status, since education can only in part overcome this problem. In fact the reason why educated females have thinner melanomas could be that they have a different pattern of sun exposure compared to men. Male gender has already been reported to be an independent risk factor for thick CM as well as living alone[21,22]. Part of the observed increase in CM incidence over the last 30 years consists in a large part of very thin CM, which could potentially be promoted by sun exposure. However this is confounded by increased awareness and screening over the last 30 years. There is a need to understand what triggers aggressive CM. Indeed, nodular melanomas are already known to be less related to sun exposure compared to superficial spreading types[3].It is important to notice that sunny holydays are not necessarily associated with sunbathing and and sunburns, well know risk factors for melanoma[23]. In fact sun exposure during hot hours and residence in tropical countries in youth, that are proxy for sunbathing and sunburns more than sunny holidays, were not found to be associated with CM prognosis. 

 Our data confirmed a greater frequency of SSM in patients going on regular holidays in the sun. However, adjusting for histology, the effect of UV exposure on CM thickness and recurrence remained. 

A potential bias for the effect of sun exposure on thickness could be screening bias, We took into account of this looking at grade of clinician and season of diagnosis. In fact subjects who had regular holidays in the sun before diagnosis were more prone to seek dermatological screening as in view of their sun seeking behaviour or because having naked skin in summer made them more aware of potentially malignant lesions. However frequencies of visits to dermatologists were similar in exposed and unexposed and all the analyses were adjusted for grade of clinician. Furthermore season of diagnosis, was not significantly associated with Breslow thickness and CM diagnosed during summer months were more frequent among patients with no holidays, which does not suggest a screening bias. 

We know that skin awareness and going on holidays are linked with education, but sunny holidays were associated with thickness and melanoma recurrence adjusting for educational status and skin awareness indicators. 

Another selection bias could be that patients who enjoy outdoor sun exposure are more likely to be in good health, compared to patients who are older and/or with comorbidities who may have a higher chance of relapse. However the two exposure groups have similar initial stage of disease. In order to avoid time related biases, such as immortal bias, we started the analysis from the time of the questionnaire. It is still possible that other unknown protective factors in sun seeking individuals explained the improved survival. 

Despite the fact that there are sources of bias that we were not able to fully address, we believe that our study shows several intriguing results that should be further investigated and are in agreement with previous epidemiological publications un sun exposure indicators and melanoma survival[4][24][25] It is also reassuring that results on recurrence are in agreement with results at baseline: sunny holidays are inversely associated with worse prognostic factors (thickness and ulceration) and recurrence. Furthermore dose-response relationships are confirmed. Moreover, if major confounders had biased results, we should have obtained similar results with other indicators of UV exposure, such as sun exposure during peak hours and sunbed use, but it was not the case: only sunny holidays were found to be inversely associated with CM prognosis. 

Solar radiation is a well-established skin carcinogen so the interpretation of the protective effects of sun exposure on melanoma needs to be made with caution. These results need to be confirmed by larger studies with measurements of Vitamin D serum levels in summer and winter as well as data on Vitamin D receptor genotypes. In the meantime, there is a need to be aware of the risk of Vitamin D deficiency in melanoma patients and we recommend measuring Vitamin D levels during follow up and offer supplementation if necessary[26].[19,27] 
