# Correction: Sunny Holidays before and after Melanoma Diagnosis Are Respectively Associated with Lower Breslow Thickness and Lower Relapse Rates in Italy

**DOI:** 10.1371/journal.pone.0101732

**Published:** 2014-07-03

**Authors:** The PLOS ONE Staff

The graphs for Figure 4a and 4b are incorrectly switched. Please see the corrected Figure 4 here.

**Figure 4:**
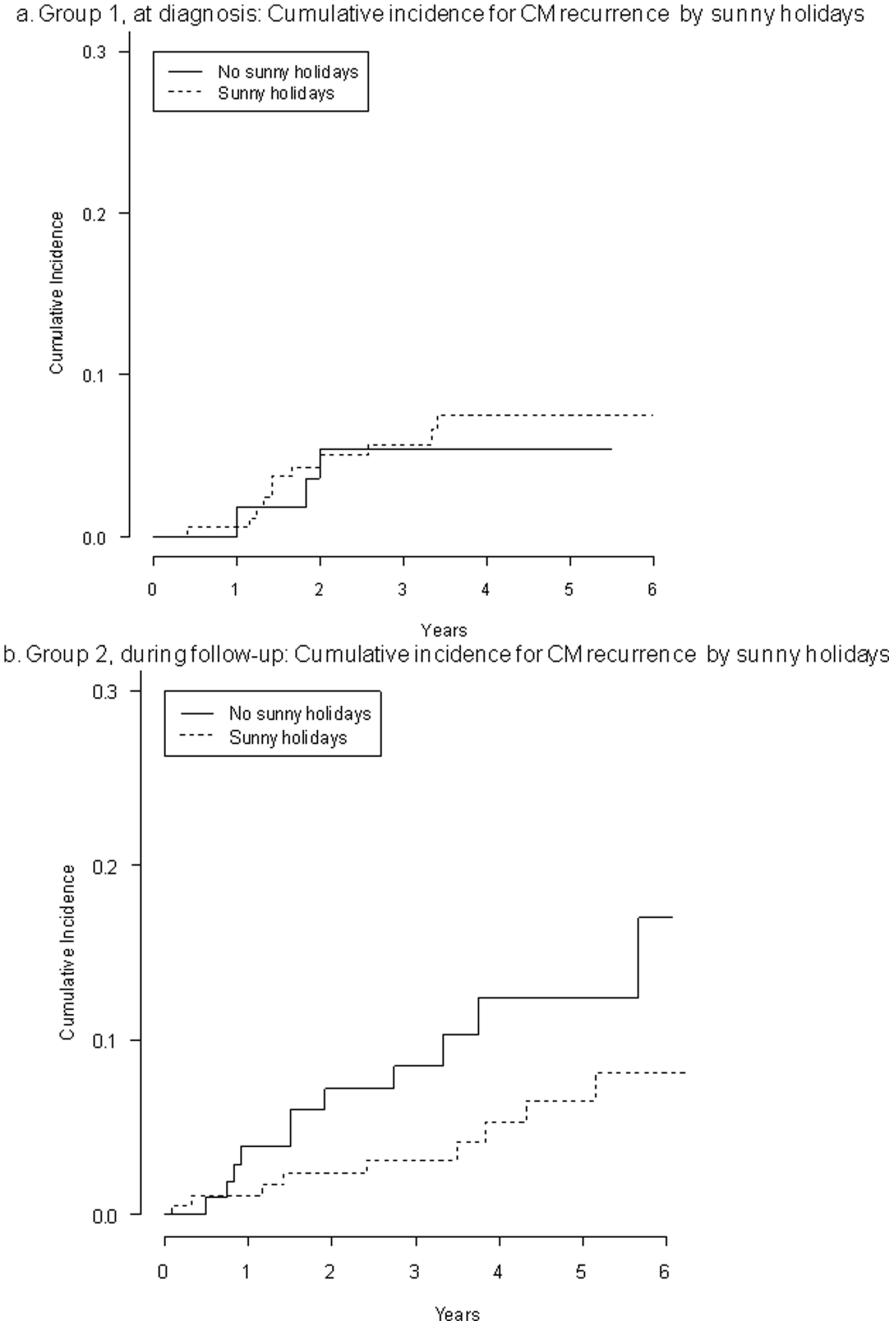
Group 1, at diagnosis: cumulative incidence for melanoma recurrence by sunny holidays (A). Group 2, during follow-up: cumulative incidence for melanoma recurrence by sunny holidays.
